# *Aspergillus terreus* Accessory Conidia Are Unique in Surface Architecture, Cell Wall Composition and Germination Kinetics

**DOI:** 10.1371/journal.pone.0007673

**Published:** 2009-10-30

**Authors:** Eszter Deak, Selwyn D. Wilson, Elizabeth White, Janice H. Carr, S. Arunmozhi Balajee

**Affiliations:** 1 Mycotic Diseases Branch, Centers for Disease Control and Prevention, Atlanta, Georgia, United States of America; 2 Infectious Diseases Pathology Branch, Centers for Disease Control and Prevention, Atlanta, Georgia, United States of America; 3 Clinical and Environmental Microbiology Branch, Centers for Disease Control and Prevention, Atlanta, Georgia, United States of America; Albert Einstein College of Medicine, United States of America

## Abstract

Infection with *Aspergillus terreus* is more likely to result in invasive, disseminated disease when compared to other *Aspergillus* species; importantly this species appears to be less susceptible to the antifungal drug amphotericin B. Unique to this species is the ability to produce specialized structures denoted as accessory conidia (AC) directly on hyphae both in vitro and in vivo. With the hypothesis that production of AC by *A. terreus* may enhance virulence of this organism, we analyzed the phenotype, structure and metabolic potential of these conidia. Comparison of *A. terreus* phialidic conidia (conidia that arise from conidiophores, PC) and AC architecture by electron microscopy revealed distinct morphological differences between the two conidial forms; AC have a smoother, thicker outer cell surface with no apparent pigment-like layer. Further, AC germinated rapidly, had enhanced adherence to microspheres, and were metabolically more active compared to PC. Additionally, AC contained less cell membrane ergosterol, which correlated with decreased susceptibility to AMB as determined using a flow cytometry based analysis. Furthermore, AC exhibited surface patches of β1-3 glucan, suggestive of attachment scarring. Collectively, the findings of this study suggest a possible role for AC in *A. terreus* pathogenesis.

## Introduction

*Aspergillus* species remain the predominant etiological agents of invasive fungal infections among patients with hematologic malignancies and recipients of solid-organ and hematopoietic stem-cell transplants [1]. Although *Aspergillus fumigatus* accounts for a majority of cases of invasive aspergillosis (IA), *A. terreus* is emerging as a significant pathogen in certain medical centers [2]. Studies also demonstrate that *A. terreus* can cause more fulminating and disseminating disease than other *Aspergillu*s species, and is refractory to treatment with the antifungal drug amphotericin B (AMB) [3], [4].

Species within the genus *Aspergillus*, including *A. terreus*, produce asexual conidia that arise from conidiophores (phialidic conidia, PC). These PC are produced by *Aspergillus* species in the natural environment and on laboratory media but are rarely produced during infection. In addition to PC, *A. terreus* produces another type of asexual conidia denoted as accessory conidia (AC), which arise directly on hyphae. Uniquely, *A. terreus* has the ability to produce AC both in vitro and in vivo during invasive infection. Detection of these distinctive structures in body fluids and/or biopsy specimens has been considered “diagnostic” for *A. terreus* infection [4]. Production of AC by *A. terreus* isolates in vivo was recorded as early as the 1950s when investigators noted the presence of these structures in the stomach contents of a bovine fetus after mycotic abortion [5]. Raper and Fennell in their monograph on the genus *Aspergillus* speculated on the possible role of these structures in dissemination of disease [5], [6]. Clinically relevant fungi such as *Scedosporium* species, *Fusarium* species, *Paecilomyces* species, and *Acremonium* species produce adventitial unicellular forms in vivo during infection [7] and it has been postulated that the high proportion of disseminated infection due to these fungi can be attributed in part to these structures. It is not known at this time if a similar function exists for *A. terreus* AC. It has also been hypothesized that AC might be the source of AMB resistance in this organism. The only previous study comparing the susceptibility of *A. terreus* AC with that of PC found that both cellular forms exhibited similar susceptibility to AMB [8].

Thus, little is known about the function and role of AC in *A. terreus* biology and its correlation with pathogenesis and AMB drug resistance. The present study was therefore designed with the following objectives: (1) to estimate AC production of a large number of *A. terreus* isolates recovered from both clinical and environmental samples, (2) to examine morphological differences between AC and PC, (3) to evaluate germination kinetics and metabolism of AC and PC, (4) to study differences in AMB susceptibilities of AC and PC using a flow cytometry based method, and finally (5) to analyze ergosterol content and β-glucan surface exposure of AC. We demonstrate that *A. terreus* accessory conidia are phenotypically distinct, structurally, in cell wall composition, and in in vitro antifungal susceptibility, and as such may contribute to the virulence of this organism.

## Methods

### Production of AC by *A. terreus* isolates from diverse sources

One hundred *A. terreus* isolates recovered from the CDC culture collection were tested for AC production. Of these, 45 isolates were recovered from clinical samples (both colonizing; i.e. clinical isolates that were recovered from sputum, tracheal aspirates, or skin samples, and invasive sites) and 55 isolates were recovered from environmental sampling (soil, air sampling, leaf litter, etc.). All isolates were cultured on Sabouraud dextrose agar plates (SDA) and incubated at 37°C for three days after which phialidic conidia were harvested in phosphate-buffered saline (PBS), and 1,500 µl of the conidial suspension (5×10^5^ cells/ml) was transferred to 40 ml Sabouraud dextrose broth (SDB) with 0.1% Tween 20 (SDB Tween) [8]. After 5 days of incubation at 28°C in SDB Tween, the production of AC by each isolate was determined microscopically.

For all assays described below, *A. terreus* isolates CLF 29 and CLF 52, recovered from an environmental and a clinical sample respectively, were employed.

### Conidial phenotypes by scanning electron microscopy (SEM) & transmission electron microscopy (TEM)

For SEM and TEM studies, *A. terreus* isolates CLF 29 and CLF 52 were sub-cultured on SDA and in SDB Tween (as described above) for production of PC and AC respectively. To prepare PC inocula, *Aspergillus* colonies were gently probed with a loop and the resultant conidia were suspended in SDB. Accessory conidia were harvested by vortexing the fungal growth and filtering through Whatman 4 filter papers for removal of contaminating hyphal fragments. For SEM, PC and AC were fixed in 5% glutaraldehyde in cacodylate buffer (0.067 mol, pH 6.2) overnight at room temperature. Conidial samples were filtered through a 0.1 µm nucleopore polycarbonate filter, placed on top of a 13 mm glass cover slip, and incubated in a chamber for final dehydration using acetone vapors. The glass cover slip with the filter attached was then mounted on an aluminum stub with silver paint and sputter coated with gold. Finished samples were observed with a FEI XL 30 ESEM (FEI Co., Hillsboro, OR).

For TEM, PC and AC were fixed in a 2% gluteraldehyde solution for one hour, followed by incubation overnight in EM buffer (0.01 M phosphate) at room temperature. Conidia were transferred into 1% osmium tetroxide for an hour to provide image contrast, dehydrated in a graded series of ethanol, after which Araldite 502 resin (Ted Pella, Inc) plastic infiltration was performed in a series of Araldite 502 resin and propylene oxide until conidia were in 100% Araldite 502 resin. The conidia in resin were placed into molds and incubated in a 60°C–70°C oven for 18–24 hours. Sections of 60 nm or less were cut using a Diatome diamond knife on a Leica Ultracut UCT and placed onto a copper grid. Grids were washed in uranyl acetate for 2 minutes, followed by lead citrate for 6 minutes, and were then examined in a Philips Techai G2 TEM.

### Germination and adherence of accessory and phialidic conidia

For evaluation of germinating potential of the conidial types, PC and AC were obtained from *A. terreus* isolates CLF 29 and CLF 52 as described above. One milliliter of SDB containing 1×10^6^conidia was transferred to a 24 well plate and incubated at either 30°C or 37°C for 2, 4, 6, 8, 12, and 22 hours. Germination of both conidial forms at different timepoints was assessed by light microscopy.

A microsphere adhesion assay was performed to assess relative adhesive properties of both conidial forms [9]. In brief, PC and AC were each resuspended in 0.1 mol/liter KNO_3_ solution (pH 6.5) and mixed with latex particles (0.8 µm; Sigma) at a ratio of 20∶1. The number of microspheres adherent to each conidium was counted using light microscopy in at least 20 different fields.

### Assessment of metabolic activity of the conidial forms

Metabolic activity of both conidial forms was assessed using the colorimetric indicator 2,3-bis(2-methoxy-4-nitro-5-sulfophenyl)-5-[(phenylamino)carbonyl]-2*H*-tetrazolium hydroxide (XTT) with an electron-coupling agent, menadione [10]. *A. terreus* AC and PC from isolates CLF 29 and CLF 52 were harvested as described above. XTT (Sigma) was diluted to 1 mg/ml in warm sterile saline. Menadione (Sigma) was prepared in acetone at a concentration of 200 mM. XTT-menadione solution was prepared by mixing 1 ml of XTT (1 mg/ml) with 200 µl diluted menadione in 20 ml of PBS. Isolated conidia were diluted to 5×10^6^ cells/ml in XTT-menadione solution, 200 µl plated in 96-well flat bottom plate, and incubated at 37°C in the dark. Optical Density (OD) was read at 0, 0.5, 1, 2, 3, 4, 5, 6, and 7 hours at 490 nm in a spectrophotometer plate reader. Wells with XTT alone were used as background controls. The OD of the well with XTT alone (background) was subtracted from the OD of well with AC or PC with XTT at every time point. Experiments were performed in triplicate.

### β1-3-glucan exposure on conidial forms

For assessing surface β-glucan exposure of both conidial forms, *A. terreus* AC and PC from isolates CLF 29 and CLF 52 were harvested as described above and resuspended in 1X PBS (plus 0.01% Tween) along with *A. fumigatus* (ATCC1022) conidia as control. Monoclonal mouse IgG antibody to β1-3-glucan (Biosupplies Australia, Parkville, Australia) was added at 1∶20 concentration and conidia were incubated on ice for 45 minutes. After washing with 1X PBS (plus 0.01% Tween), conidia were resuspended in goat anti-mouse Alexa Fluor 594 conjugated antibody (Molecular Probes) diluted 1∶250 in 1X PBS/plus 0.01% Tween, and incubated on ice for 45 minutes. Conidia were washed with 1X PBS/0.01% Tween, resuspended in Fluoromount G (Electron Microscopy Sciences), mounted onto slides, and examined under a fluorescent microscope (Zeiss Axiovert 25).

### Measurement of Cell Membrane Ergosterol Levels and susceptibility to amphotericin B

Cell membrane ergosterol levels were analyzed by a previously published method with slight modifications [11]. Conidial suspensions of both AC and PC from isolates CLF 29 and CLF 52 were harvested as described above and suspended in 150 µl of 25% alcoholic potassium hydroxide solution and mixed for 2 minutes, followed by incubation at 85°C for 2 hours. Sterols were extracted from each sample by adding 60 µl of sterile water and 150 µl of *n*-heptane, mixing for 2 minutes, and allowing the layers to separate for 30 minutes at room temperature. The *n*-heptane layer was recovered for spectrophotometric analysis at 230 and 281.5 nm with a Beckman spectrophotometer, and the ergosterol content was calculated based on the absorbance and wet weight of the initial pellet as previously described [12].

Both conidial forms were assessed for their susceptibilities to AMB using the FUN 1 based assay as described previously and the CLSI microbroth dilution method [13]. For the flow cytometry based assay (FCM), PC and AC were obtained from *A. terreus* isolates CLF 29 and CLF 52, and 200 µl of the conidial suspension (5×10^6^ cells/ml SDB) was dispensed into polystyrene round-bottom FACS tubes. Two hundred microliters of AMB (Sigma) (diluted in SDB) were added to yield final drug concentrations of 2 µg/ml to 64 µg/ml [14] and the tubes were incubated at 37°C for 4 hours. FUN-1 was added to a final concentration of 0.5 µmol, incubated in the dark for 40 minutes at 37°C with shaking, and acquired in FACScan cytometer with data analyzed using CellQuest software.

The CLSI microbroth method was performed as previously described with a slight modification wherein both the conidial forms were counted using a hemocytometer before the conidial inocula (5×10^5^) were dispensed into microtiter wells containing serial twofold dilutions of AMB, and plates were incubated for 48 h at 35°C. MIC was defined as the lowest concentration of drug that produced 100% reduction in the optical density at 550 nm (OD_550_) relative to the growth control.

In these studies, the Student T-test was used to determine statistical significance.

## Results and Discussion

### Both clinical and environmental *A. terreus* isolates produce accessory conidia

**Figure 1:**
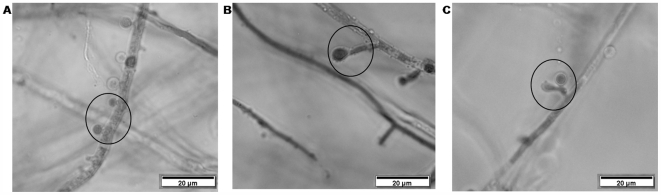
*Aspergillus terreus* Accessory conidia production. Light microscopic images of accessory conidia recovered from *A. terreus* isolates IBT 12713 (A), P12 (B), E5 (C) demonstrating globose (A) and club-shaped (B) and in pair (C) phenotypes. Scale bars represent 20 µm.

Although it has been previously documented that *A. terreus* can produce AC, it was not known if all strains within this species can produce these structures or if there is variability in quality and quantity of AC production between *A. terreus* strains recovered from clinical and environmental origin. In this study, we examined 100 *A. terreus* isolates from both clinical and environmental samples for their ability to produce AC in vitro. We found that although there were differences in the number of AC that each isolate produced, all *A. terreus* isolates, regardless of their origin, produced these structures (data not shown). We further speculated that the ability to produce AC may better facilitate *A. terreus* to establish infection, and thus isolates recovered from clinical samples may have the potential to produce more AC than isolates recovered from environmental samples. Results of our study demonstrate that isolates from clinical samples produced on average 2.3×10^5^ conidia/ml while isolates from environmental samples produced on average 1.9×10^5^ conidia/ml - thus there was no correlation between the origin of the isolate and AC production. Interestingly, our study shows a wide variability in the size and shape of AC ranging from globose (Figure 1A), to ellipsoidal, to club-shaped (Figure 1B). Some isolates produced AC in clusters, some in pairs (Figure 1C), and in other cases AC arose singly from hyphae (Figure 1A–B). The variation in AC morphology was strain dependent and was fairly consistent for a given strain. Thus, this study demonstrates that all the tested *A. terreus* isolates regardless of origin (clinical versus environmental) have the ability to produce AC, albeit in different quantities and shapes.

### Accessory conidia have distinct cell wall architecture

**Figure 2:**
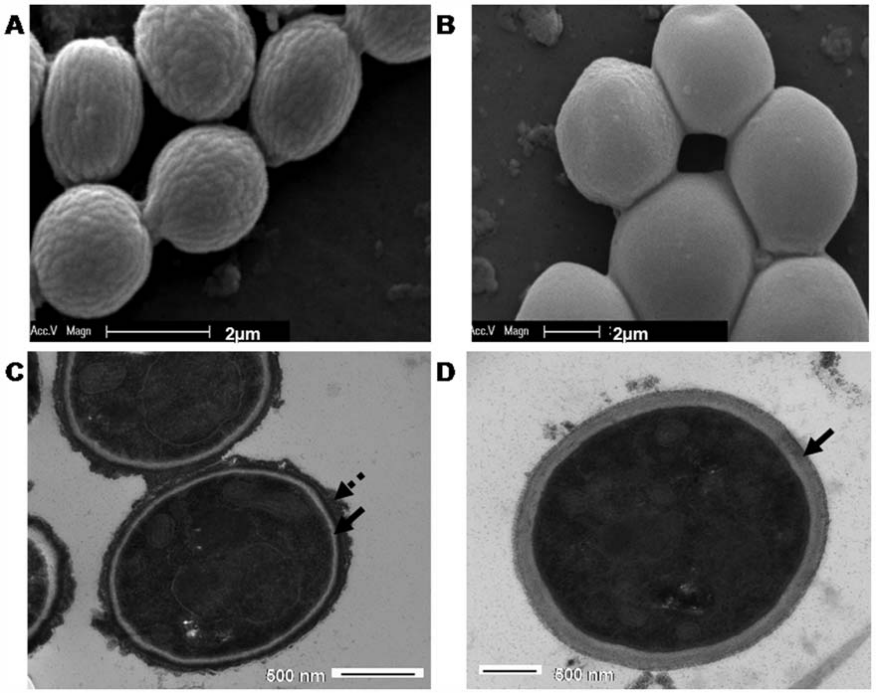
Cell wall architecture of phialidic and accessory conidia of A. terreus. Comparative scanning electron microscopy (A and B) and transmission electron microscopy (C and D) of *A. terreus* phialidic conidia (A and C) and accessory conidia (B and D). Conidia were harvested and fixed in 5% glutaraldehyde for microscopy. Dashed arrow indicates melanin like layer and solid arrow indicates the cell wall (C and D); 10–15 conidia were imaged, p = 0.0003.

Little or nothing is known thus far about the cell wall architecture of the PC and AC of *A. terreus* and to address this, we performed SEM and TEM imaging studies on both these conidial forms with the PC of *A. fumigatus* as a control. Previous studies have revealed that the *A. fumigatus* conidial cell wall has an outermost melanized layer consisting of interwoven rodlet fascicles and other hydrophobins, giving the conidia their green, echinulate morphology and rendering them hydrophobic [15]. Homologous structures have also been demonstrated in *A. oryzae*, *A. niger*, and *A. nidulans* [9]. In keeping with these studies, our study revealed that *A. fumigatus* PC were 2–4 µm in diameter, and had an ornamented surface with distinct protuberances giving it an echinulate morphology (data not shown), whereas the surface of *A. terreus* PC had a striated outer surface with rod shaped structures that were arranged closely on the surface; PC were 2–4 µm in diameter (Figure 2A). In contrast to PC of both these aspergilli, *A. terreus* AC were bigger, about 4–7 µm in diameter exhibiting a smooth conidial surface with no rod shaped structures or protuberances evident (Figure 2B).

Beneath the melanized conidial surface of *A. fumigatus* lies the cell wall that is composed of β1-3 glucan, β1-3 glucan synthase, chitin, chitin synthases, and galactomannan, among other cell associated molecules [15]. In this study, TEM analysis of both *A. terreus* and *A. fumigatus* PC revealed that the outermost cell surface layer of conidia from both species was composed of a thick, dark melanin-like layer (Figure 2C, indicated with a dashed arrow) under which a thin cell wall layer was evident (Figure 2C, indicated with a solid arrow). In marked contrast, the outermost dark, melanin-like outer layer was absent in *A. terreus* AC and a smooth, thicker non-pigmented cell wall was observed (Figure 2D, indicated with a solid arrow). Collectively, the SEM and TEM studies demonstrate that the outer surface of AC was smoother and thicker than PC (∼125±7 nm AC versus 50±3 nm PC; 10–15 conidia were imaged, p = 0.0003) and appeared to lack the dark pigmented layer characteristic of PC.

### Accessory conidia differ from Phialidic conidia in germination properties, metabolic activity and adherence

We hypothesized that differences in cell wall architecture of AC (as observed by SEM and TEM studies) may manifest as other phenotypic differences such as rate of germination and adherence properties of the conidial forms. Results of this study show that AC germinated rapidly (germ tubes were observed within 2 hours of incubation) in vitro when compared to PC (no germ tubes observed at 2 hours) at both temperatures tested. We did not determine the ratio of germinated to ungerminated conidia of both cellular forms for this study. Germination experiments in this study were in vitro observations, performed with only two representative *A. terreus* isolates, and will need to be confirmed with more isolates and in other environments. If confirmed, the ability of AC to germinate rapidly into tissue invasive hyphal forms may be a virulence factor for *A. terreus*, given that exit from conidial dormancy and subsequent germination is essential for the establishment of infection [4], [16].

In keeping with the altered cell surface phenotype of AC when compared to PC, we found that AC were significantly more adherent to microspheres than PC (26% of AC adhered versus 16% of PC; p value  = 0.01) reiterating the cell surface associated differences between these conidial forms. *Aspergillus nidulans* rodletless mutant strains are more adherent to microspheres than wild-type strains, resulting in changes in physico-chemical properties of the conidia, including reduced hydrophobicity and decreased polarization [9]. In contrast, no differences in adherence were observed between the rodletless mutants and wild type isolates of *A. fumigatus*. However, studies with *A. fumigatus* rodlet A mutants and germ tubes that have lost the hydrophobin layer indicate other changes in cell surface receptor expression that affect conidial binding potential to certain surfaces [17]. Thus, the smooth surface of *A. terreus* AC described in this study and its altered adherence phenotype may reflect a lack of surface hydrophobins. Additionally, studies analyzing differences in adherence of both conidial forms to other surfaces and the cell wall composition of AC are warranted to substantiate this observation.

**Figure 3:**
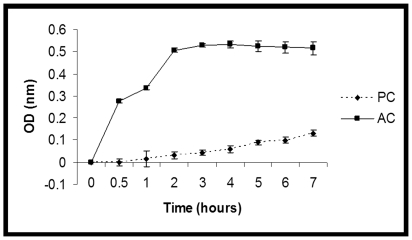
Metabolic activity of phialidic conidia and accessory conidia using the XTT assay. XTT absorbance (OD_490_) of phialidic (dashed line) and accessory conidia (solid line) was measured at 0, 0.5, 1, 2, 3, 4, 5, 6, and 7 hours after subtraction of background OD (OD of wells with XTT alone). Error bars represent standard error of experiments performed in triplicate.

Next, we compared the metabolic activity of both conidial forms using the viability dye XTT. This dye is reduced to a colorimetric formazan product in the presence of metabolically active cells and has been used to quantify metabolic activity and viability of *Aspergillus* isolates [18]. Results of the present study showed that the ACs were metabolically active as early as thirty minutes as evidenced by an increase in XTT-formazan production; OD of the ACs continued to increase rapidly within two hours after which absorbance reached a plateau (Figure 3). In contrast, at two hours the PCs showed only a slight increase in XTT-formazan production with the XTT conversion rate being slower than compared to ACs even after seven hours of incubation (the last time point tested in this study). This finding was in keeping with another study where the XTT conversion of *A. terreus* PC was lower when compared to *A. flavus* and *A. fumigatus* and was attributed to poor metabolic activity at this early time point (Walsh et al, 2007). Collectively, our study suggests (at least with the strains tested) that ACs have robust metabolic activity when compared to PCs and that this metabolic activity begins earlier in ACs.

Our study demonstrates that AC, in comparison with PC, can become metabolically active, germinate rapidly and are more adherent. In vitro experiments demonstrated that these AC can be readily dislodged from hyphae (data not shown). Thus, it is tempting to speculate that during infection in vivo these structures, once pinched off from hyphae at the site of infection, can disseminate to other body sites where they can establish infections swiftly by virtue of their adherence and germination properties. In keeping with this premise, previous studies have shown that *A. terreus* isolates have a propensity to cause more disseminated and fulminating infections than other *Aspergillus* species [19].

### Accessory conidia have less cell membrane ergosterol content and higher MICs to AMB

Our analysis of ergosterol levels by previously described methods [12] demonstrated that AC had lower cell membrane ergosterol content when compared to PC (32.3% PC versus 10.1% AC for CLF 52; 37.6% PC versus 4.8% AC for CLF 29; p = 0.01; representative of three separate experiments). Since it has been shown that cell membrane ergosterol content may correlate with antifungal drug susceptibility to AMB [4], [20], we tested the AMB drug susceptibilities of both conidial forms by a FUN 1 based FCM assay [14]. Results of this assay showed that both *A. terreus* AC had AMB MICs that were higher than MICs of PC (AC mean MIC  =  2 µg/ml; PC mean MIC  = 8μg/ml). These results differ from that of a recent study that found no difference in AMB MICs of both conidial forms [8] and may be in part due to the differences in methodologies: we employed a fluorochrome based FCM assay while Lass-Flörl and co-workers generated MICs with the CLSI M38A microdilution method. Another reason that our study results may differ from the previous study could be that we assayed MICs at 4 hours versus the CLSI study that requires 48 hours of incubation before the results can be read. Since ACs had a propensity to be metabolically active within two hours and PCs did not, we also generated AMB MICs employing the CLSI broth microdilution method [13] to exclude the effect of differences in metabolic activity of the cellular forms on MIC interpretation at an early timepoint employing a FCM method. Although the AMB MIC generated by the CLSI method was at least two fold lower than MIC generated by the FCM method, both ACs had at least four fold higher MICs when compared to PC (mean MIC  = 4 µg/ml for AC versus 1 µg/ml for PC). Thus in our study, ACs had a lower susceptibility to AMB when compared to PCs by both the FCM and the traditional CLSI based method. The finding that AC have lower cell membrane ergosterol that correlates with higher MICs to AMB may be in part why *A. terreus* isolates fail to respond to AMB therapy in vivo [21] and needs further investigation with more isolates.

### Differential binding of the β1-3 glucan antibody to the two conidial forms

**Figure 4:**
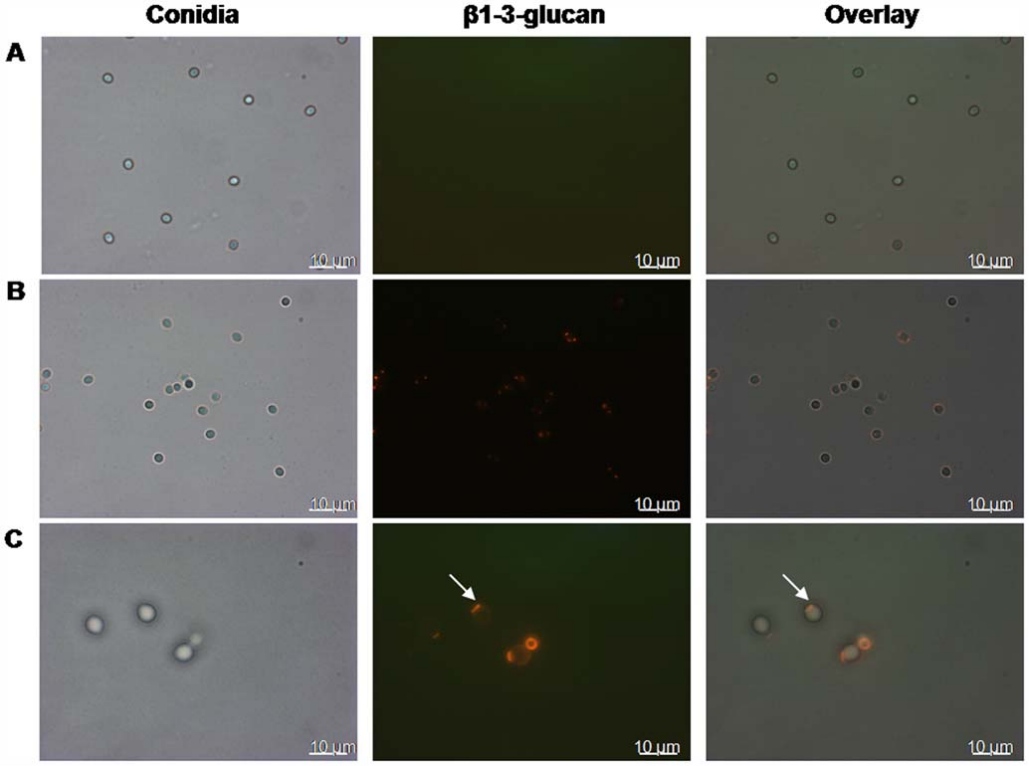
β1-3-glucan expression on phialidic conidia and accessory conidia. Staining for β1-3-glucan surface exposure on *A. fumigatus* conidia (A), *A. terreus* phialidic conidia (B) and *A. terreus* accessory conidia (C) was performed and evaluated by fluorescence microscopy. Arrows denote the β1-3-glucan staining on accessory conidia.

The cell wall architecture of the asexual conidia of *A. terreus* has never been studied, and that of *A. fumigatus* is only now being understood. Recent studies with *A. fumigatus* resting conidia have demonstrated that low concentrations of β1-3 glucan are accessible on the surface and only conidial germination unmasks the β1-3 glucan surface moieties [22]. We found that resting *A. terreus* phialidic conidia (similar to *A. fumigatus* – Figure 4A) demonstrate low β1-3 glucan labeling (Figure 4B) indicating that, as with other fungi, a cell wall layer may be enveloping the β1-3 glucan moiety, thus efficiently masking *A. terreus* conidia from immune recognition [22], [23], [24], [25]. In contrast, while the surface of the AC was also largely shielded from β1-3 glucan exposure (as observed by low β1-3 glucan labeling), a patch of the AC was intensely labeled with β1-3 glucan antibody indicating significant levels of β1-3 glucan at this site (Figure 4C indicated with an arrow). We speculate that this region would have been previously attached to the hyphae and therefore represents the attachment scar. The question arises as to why AC exhibit scarring while PC do not. The answer may lie in mode of attachment of these structures on hyphae. Phialidic conidia are never borne directly on hyphae, but rather are pushed out or bud from within the phialide, the flask shaped structures comprising the reproductive zones of the conidiophore. In contrast, AC arise directly from hyphae, and this type of attachment may result in prominent β1-3 glucan scars when AC are pinched off the hyphae. Regardless, we demonstrate for the first time the existence of unique β1-3 glucan rich patches on AC that may represent glucan rich attachment scars.

In the pathogenic yeast *Candida*, it has been shown that bud and birth scars are rich in surface β1-3 glucan, and that this glucan exposure appears to be sufficient to activate Dectin-1 (a β-glucan specific receptor) and trigger potent antifungal inflammatory responses in macrophages [25]. In *A. fumigatus*, genetic deletion of a polyketide synthase involved in pigment biosynthesis (PksP) results in surface exposure on resting conidia, rendering the PksP mutants vulnerable to efficient macrophage phagocytosis [24], [26]. Further, given that β-glucans can trigger inflammatory responses in macrophages, it would be noteworthy to analyze the macrophage cytokine response after exposure to AC and PC in addition to assaying for differences in phagocytosis, and experiments are under way in our laboratory to test both these hypotheses.

In summary, this study presents preliminary evidence indicating a possible role for accessory conidia in *A. terreus* pathogenesis during infection. Accessory conidia exhibit candidate virulence factors – adherence, excellent viability, rapid germination potential. Additionally, ACs have lower ergosterol content rendering them less susceptible to damage due to the antifungal drug AMB, furthering their pathogenic potential. Since most of these experiments were performed with one or few representative isolates, studies with more isolates and other detailed examinations are needed to understand the cell wall composition of these specialized structures and their interaction with immune cells.

### Disclaimer

The findings and conclusions in this article are those of the author(s) and do not necessarily represent the views of the CDC.
